# A Recurrent Germline Mutation in the 5’UTR of the Androgen Receptor Causes Complete Androgen Insensitivity by Activating Aberrant uORF Translation

**DOI:** 10.1371/journal.pone.0154158

**Published:** 2016-04-25

**Authors:** Nadine C. Hornig, Carine de Beaufort, Friederike Denzer, Martine Cools, Martin Wabitsch, Martin Ukat, Alexandra E. Kulle, Hans-Udo Schweikert, Ralf Werner, Olaf Hiort, Laura Audi, Reiner Siebert, Ole Ammerpohl, Paul-Martin Holterhus

**Affiliations:** 1 Department of Pediatrics, Division of Pediatric Endocrinology and Diabetes, Christian-Albrechts-University Kiel & University Hospital Schleswig-Holstein, Campus Kiel, Kiel, Germany; 2 Pediatric Clinic, CH de Luxembourg, Luxembourg, GD de Luxembourg; 3 Department of Pediatrics, Division of Pediatric Endocrinology and Diabetology, University Ulm, Ulm, Germany; 4 Department of Pediatrics, Division of Pediatric Endocrinology, Ghent University Hospital and Ghent University, Ghent, Belgium; 5 Department of Medicine III, University Bonn, Bonn, Germany; 6 Department of Pediatrics, Division of Experimental Pediatric Endocrinology, University Lübeck, Lübeck, Germany; 7 Hospital Vall d'Hebron, Vall d’Hebron Research Institute (VHIR) & Centre for Biomedical Research Network on Rare Diseases (CIBERER), Barcelona, Spain; 8 Institute of Human Genetics, Christian-Albrechts-University Kiel & University Hospital Schleswig-Holstein, Campus Kiel, Kiel, Germany; CNRS UMR7275, FRANCE

## Abstract

A subset of patients with monogenic disorders lacks disease causing mutations in the protein coding region of the corresponding gene. Here we describe a recurrent germline mutation found in two unrelated patients with complete androgen insensitivity syndrome (CAIS) generating an upstream open reading frame (uORF) in the 5’ untranslated region (5’-UTR) of the androgen receptor (*AR*) gene. We show in patient derived primary genital skin fibroblasts as well as in cell-based reporter assays that this mutation severely impacts AR function by reducing AR protein levels without affecting *AR* mRNA levels. Importantly, the newly generated uORF translates into a polypeptide and the expression level of this polypeptide inversely correlates with protein translation from the primary ORF of the *AR* thereby providing a model for *AR*-5′UTR mediated translational repression. Our findings not only add a hitherto unrecognized genetic cause to complete androgen insensitivity but also underline the importance of 5′UTR mutations affecting uORFs for the pathogenesis of monogenic disorders in general.

## Introduction

Post-transcriptional control of gene expression is considered an important mechanism for cells to quickly respond to internal and external stimuli. The untranslated regions (UTRs) at both ends of an mRNA play thereby an important role. While the 3′UTR has been extensively studied as a major place of regulation e.g. through microRNA mediated mRNA degradation and translational inhibition [1], translational regulation through the 5′UTR is a field that more recently came into focus. 5′UTRs are the sites where ribosomes enter and scan the mRNA for a suitable translational start codon at which the translation initiation complex becomes assembled. This scanning can be influenced by secondary structures of the mRNA as well as the presence of upstream open reading frames [2]. The latter are cis-regulatory sequences in the 5’-UTR of eukaryotic mRNAs defined by a start codon and an in frame stop codon, located either upstream of or overlapping with the main initiation codon of the gene. uORFs can reduce translation of the downstream encoded protein by hindering ribosome progression or sequestering functional pre-initiation complexes [3]. uORF mediated post-transcriptional regulation of gene expression has been associated with stress response and mutations changing the uORF have been implicated in the etiology of human diseases [3–6].

The 5′UTR of the androgen receptor has several features characteristic for translational regulation: its 1,115 nucleotides (nt) largely exceed the typical length of 100–220 nt across species and it is GC rich, thus prone to secondary structures. The AR belongs to the nuclear receptor superfamily of transcription factors. It is activated by its ligand dihydrotestosterone (DHT) which induces a conformational change of the AR and its translocation into the nucleus. Uncontrolled high AR-activity can lead to prostate cancer [7]. Too low AR-activity on the other hand can cause the monogenic condition androgen insensitivity syndrome (AIS), defined by the inability of the androgen receptor to properly respond to androgens i.e. DHT [8]. AR-activity is essential for male sex development and individuals with AIS although having a male chromosome set and normal male serum testosterone levels develop with a partial or complete female phenotype. AIS is a common cause of disorders of sex development (DSD) and can be divided in three major subgroups: complete AIS (CAIS) with no residual AR-function characterized by female external genitalia, partial AIS (PAIS) with a variably impaired AR causing incomplete virilisation, and mild AIS (MAIS) presenting with normal male external genitalia but infertility [9, 10]. In over 85% of cases, CAIS is explained by loss of function mutations affecting the coding region of the *AR* gene, while this applies only to roughly one-third of PAIS cases [11, 12].

Androgen signaling has been extensively studied mainly in prostate cancer. Comparatively little is known about the regulation of *AR* mRNA stability and translation. In 1994, Mizokami et al. [13] showed, using reporter assays, that parts of the *AR*-5′UTR are essential for induction of translation but not transcription. They hypothesized that mutations in the 5′UTR of the *AR* could be one of the reasons for androgen insensitivity due to reduced AR translation without affecting AR mRNA levels. However, up to now, no such mutation has been described.

Here we present two unrelated 46,XY girls diagnosed with CAIS but lacking mutations in the coding region of the *AR*. Genomic next generation sequencing (NGS) of the complete *AR* locus revealed the same c.-547C>T germline mutation in the *AR*-5’-UTR of both patients. We show both *in vitro* and *in vivo* that this mutation introduces a translated uORF in the 5′UTR of the AR and results in a reduced AR protein expression without affecting *AR* mRNA accumulation.

## Results

### Two individuals presenting with complete androgen insensitivity but lacking mutations within the coding region of the *AR*

Patient 1 was born at term with female external genitalia. At the age of two weeks, an inguinal hernia on the left side was detected. Ultrasound examination suggested a testis within this hernia and a second testis at the internal orifice on the right. Lack of a uterus in combination with a blind ending vagina was detected and confirmed during surgical exploration. Chromosomal analysis revealed a 46,XY karyotype. Laboratory testing showed a massively elevated plasma testosterone concentration of 1,065 ng/dL (normal male range 14–363 ng/dL) together with an LH level of 2.2 IU/L (normal male range <0.3–2.5 IU/L). Therefore, the phenotype of the patient and the hormonal picture indicated the clinical diagnosis of CAIS. However, subsequent Sanger sequencing of the complete *AR* coding gene region including the intron-exon boundaries did not reveal any mutation. Further laboratory data are listed in Table 1.

**Table 1:** Additional data on index patient 1.

Analysis performed	findings
sequencing of the *AR* gene	no mutation within coding sequence (CDS) and intron/exon boundaries
sequencing of the *SRD5A2* gene	no mutation within CDS and intron/exon boundaries
sequencing of the *NR5A1* gene	no mutation within CDS and intron/exon boundaries
sequencing of the *HSB17B3* gene	no mutation within CDS and intron/exon boundaries
array-CGH	unsuspicious
gonadal histology after gonadectomy (2.75 years)	immature tubuli seminiferi
	TSPY+ germ cells in about 50% of the tubuli
	rare OCT3/4+ germ cells
	rare Sertoli cells, rare Leydig cells
	interstitial edema

Patient 2 was a term newborn with female external genitalia. In the second year of life, inguinal hernias occurred at both sides and were surgically repaired. At the age of six years, due to tomboyish behavior the mother insisted on chromosome analysis revealing a 46,XY karyotype. Subsequently the patient was transferred to a Pediatric Endocrinology Center. An hCG test (5,000 IU hCG/m2 i.m., once) was performed and indicated normal Leydig cell function with a rise of testosterone from 28 ng/dL to 238 ng/dL after 72 h. Surgical exploration confirmed the absence of a uterus and ovaries. Histology of gonadal biopsies showed immature testicular tissue with strong immunohistochemical expression of α1-inhibin. Therefore, the patient’s phenotype in conjunction with the laboratory data indicated the clinical diagnosis CAIS. However, no mutation was detected in the *AR* coding gene region including the intron-exon boundaries. During her most recent visit at the age of 13.6 years, she presented with a breast Tanner stage B4 and a plasma estradiol concentration of 18.9 ng/L (normal for B3 in 46,XX girls). No clinical signs of virilisation, i.e. clitoromegaly, were present despite a very high plasma testosterone of 693ng/dL (upper male range). Further laboratory data are listed in Table 2.

**Table 2:** Additional data on index patient 2.

Analysis performed	findings
sequencing of the *AR* gene	no mutation within CDS and intron/exon boundaries
sequencing of the *NR5A1* gene	no mutation within CDS and intron/exon boundaries
sequencing of the *HSB17B3* gene	no mutation within CDS and intron/exon boundaries
hCG test (8.75 years)	5.000 IU hCG/m^2^ i.m: testosterone rise from 28 ng/dL to 238 ng/dL; DHT rise from 8ng/dL to 33ng/dL
	testosterone / dihydrotestosterone ratio 7.21 (normal)
Gonadal histology after orchidopexy (7.66 years)	strong immunohistochemical expression of a1-inhibin
12.5 years	LH: 26.33 IU/L

### Identification of a 5′UTR mutation in the *AR* in both patients

**Fig 1:**
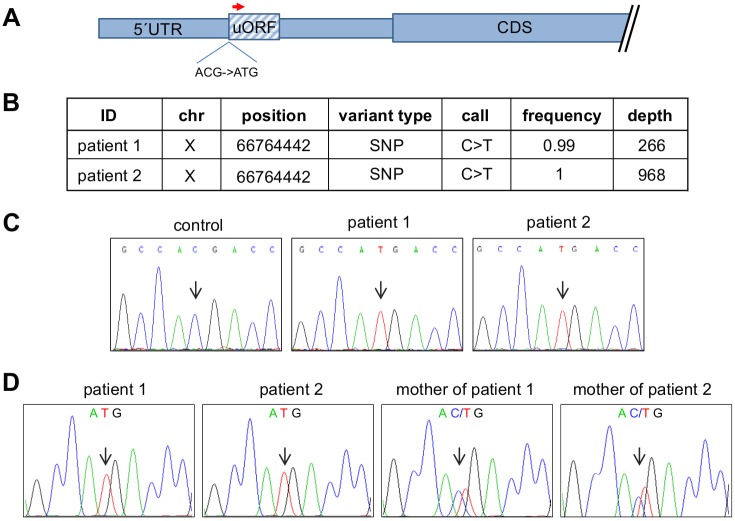
Next generation sequencing of the AR in the two index patients. A) Schematic representation of the *AR*-5′UTRs and CDS. The position of the c.-547C>T mutation and the uORF are indicated. B) A Haloplex library spanning the coding region, introns, UTRs and up and downstream sequences of the AR genomic locus was prepared from DNA of the index patients′ GF and sequenced on a MiSeq benchtop sequencer. Analysis for single nucleotide polymorphisms (SNP) and small insertion deletions (indels) was performed by the SureCall software (Agilent). Indicated is the mutation found in the 5′UTR of the *AR*. A frequency of 1 corresponds to 100% of the reads. The depth indicates the number of reads covering the indicated genomic position. C) Sanger sequencing of a male control and the index patients 1 and 2. The sequences are visualized as reverse complement strand using the Chromas Lite software and show the c.-547C>T mutation in both index patients but not in the male control. D) Sanger sequencing of blood derived DNA from both index patients as well as from the mothers of the index patients. The sequences are visualized as reverse complement strand using the Chromas Lite software and show the c.-547C>T mutation in both index patients and in the patients′ mothers in a heterozygous constellation.

We hypothesized that the CAIS-phenotype of the two patients could be due to a mutation in regulatory regions of the *AR* gene that usually escape routine Sanger sequencing. We therefore used an NGS approach to sequence the entire *AR* genomic locus in the two patients′ genital skin fibroblasts (GF) including the promoter, untranslated regions and the introns. In line with routine Sanger sequencing, NGS did neither reveal mutations in the *AR* coding sequence nor in the intron-exon boundaries. Instead, analysis for single nucleotide polymorphisms (SNP) and small insertion deletions (indels) of the NGS-data showed a hemizygous C to T mutation in both patients located in the 5′UTR of the *AR* mRNA (c.-547C>T; NM_000044.3; hg19) (Fig 1A and 1B, S1 and S2 Files). In order to exclude that the patients are related, we checked the SNP distribution in the analyzed region as well as the length of the polyglutamine repeat present in exon 1 which is located only 716 base pairs downstream the c.-547C>T mutation. SNP analysis revealed two different haplotypes (S1 and S2 Files). Furthermore the polyglutamine stretch in patient 1 consists of 24 glutamines compared to 19 glutamines in patient 2 indicating that the patients are indeed not related. We subsequently screened GF derived genomic DNA from further 25 foreskin controls without detecting the c.-547C>T mutation which was also absent in publicly available SNP databases including dbSNP and the 1000 Genome Project (hg19; release 68) The presence of the mutation was confirmed by Sanger sequencing in GF (Fig 1C) as well as peripheral blood of both patients indicating that the mutation is of germline rather than somatic origin. Consistently, the mutation was detected in a heterozygous constellation in the mothers of both patients (Fig 1D).

Context analysis of the mutation showed that the c.-547C>T mutation creates a translational initiation codon ATG imbedded in a vertebrate Kozak sequence (S1 Fig) [14]. 183 nucleotides downstream of the ATG are two consecutive Stop-codons thereby delineating a short uORF within the *AR*-5′UTR schematically depicted in Fig 1A.

### Functional analysis of the *AR*-5′UTR mutation *in vitro*

**Fig 2:**
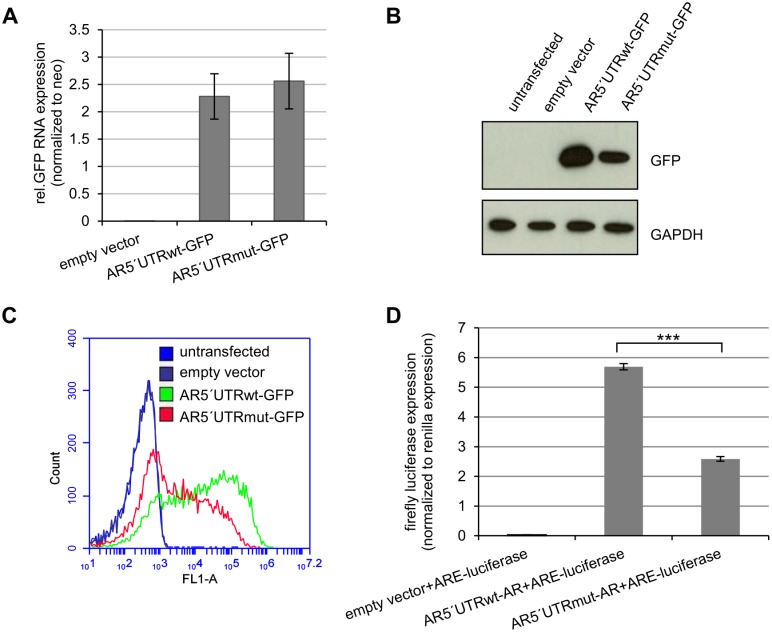
The c.-547C>T mutation in the *AR*-5′UTR reduces translation and AR activity *in vitro*. HEK293 cells were transfected with either empty vector, *AR*5′UTRwt-*GFP* or *AR*5′UTRmut-*GFP*. After 72h of transfection RNA and protein was isolated. A) Q-RT-PCR analysis of GFP mRNA. There is no significant difference between *GFP* mRNA levels of the *AR*5′UTRwt-*GFP* and the *AR*5′UTRmut-*GFP* construct (p = 0.57). *GFP* mRNA levels were normalized to the neomycin resistance (neo) expression of the vector. Experiments were performed in triplicate. B) GFP protein analysis. Cells transfected with the *AR*5′UTRmut-*GFP* construct show a reduced expression of the GFP protein compared to the wt-construct. GAPDH measurement served as loading control. The experiment was done in triplicate. One representative blot is displayed. C) FACS analysis of *AR*5′UTR-*GFP* transfected cells. FACS analysis was performed equally 72h after transfection. Cells transfected with *AR*5′UTRmut-*GFP* show less fluorescent intensity. D) Transcriptional activity of *AR*5′UTRwt-*AR and AR*5′UTRmut-*AR*. HEK293 cells were transfected with either empty vector, *AR*5′UTRwt-*AR* or *AR*5′UTRmut-*AR*. After 48h of transfection luciferase activity was measured. AR induced luciferase expression is significantly lower in *AR*5′UTRmut-*AR* transfected cells in respect to *AR*5′UTRwt-*AR* transfected cells (p***<0.001).

In order to functionally characterize the impact of the identified mutation, we cloned the entire 5′UTR of either the wild type (wt) or the mutated (mut) *AR* directly upstream of a green fluorescent protein (GFP) reporter gene and transfected the fusion constructs in HEK293 cells. Importantly, the newly generated uORF is out of frame with the primary ORF (pORF) of the *AR* as well as of the *GFP* in the *AR*5′UTR-*GFP* vector and therefore cannot create an AR-uORF-GFP fusion protein. Transcript analysis of the *GFP* reporter showed no difference in mRNA accumulation levels between the wild type and mutant *AR*5′UTR-*GFP* fusion construct (Fig 2A). In order to exclude a possible *GFP*-amplification from plasmid DNA during the RNA quantification, we included a control without reverse transcription which showed no amplification product underlining the specificity of the *GFP* mRNA measurement (S2 Fig). In contrast to comparable *GFP* mRNA levels, GFP detection by Western blot showed a reduced GFP protein expression of the mutant *AR*5′UTR-*GFP* construct as compared to the wild type construct (Fig 2B). We also performed fluorescence activated cell sorting (FACS) analysis in order to compare the fluorescent intensity between the wild type and the mutant construct on a single cell basis. This revealed a lower GFP signal intensity in cells transfected with the mutant *AR*5′UTR-*GFP* vector as compared to wild type transfected cells (Fig 2C). Therefore, we show in two independent approaches that the mutation in the *AR*-5′UTR identified in both patients is causative for the reduced GFP protein level.

We then wanted to investigate, if the mutation in the 5′UTR also reduces AR-protein function *in vitro*.

To this aim we cloned both wild type and mutant 5`UTR in front of the *AR*-CDS and transfected the corresponding plasmids into AR-negative HEK293 cells together with a plasmid containing an androgen response element (ARE) in front of a luciferase reporter gene. Firefly luciferase measurements were normalized for transfection efficiency by co-measuring Renilla luciferase expression. Cells transfected with the *AR*5′UTRwt-*AR* construct showed a mean 5.7 fold induction of AR mediated luciferase expression while this induction was strongly reduced in cells transfected with the *AR*5′UTRmut-*AR* plasmid (Fig 2D). This proves that the mutation in the AR5′UTR is responsible for a reduced transcriptional activity of the AR protein.

### Expression analysis of wildtype and mutated *AR* in vivo and in vitro

**Fig 3:**
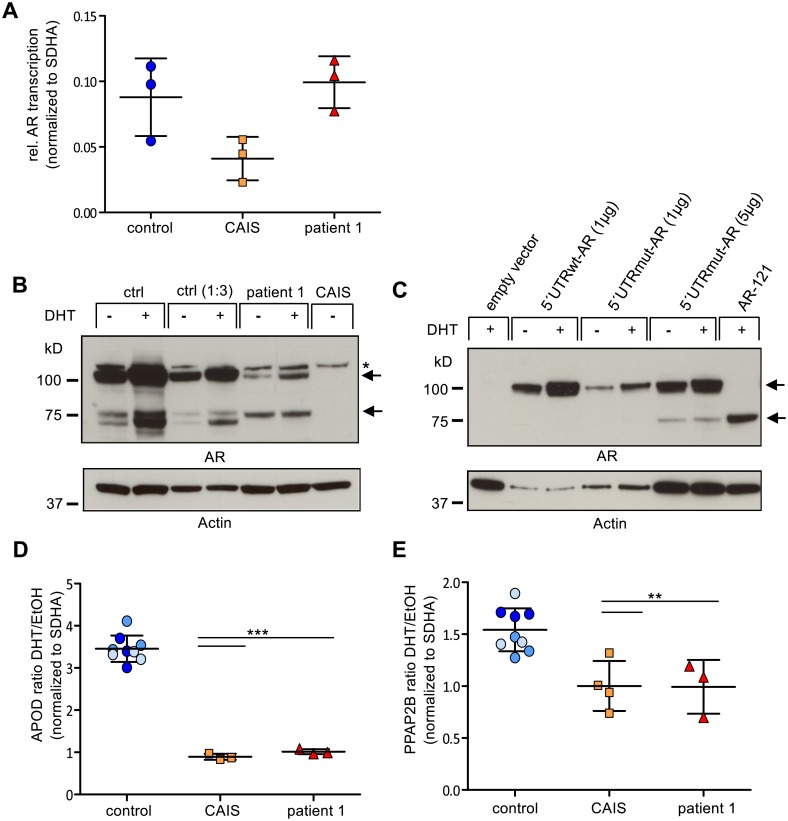
Functional and expression analysis of the AR *in vivo* and *in vitro*. A) *AR* mRNA accumulation in GF. Total RNA was extracted from GF of a male control, a CAIS patient with a documented frameshift mutation in the *AR* gene and index patient 1. Means and standard deviations of three independent experiments are shown. B) DHT-dependent AR protein expression in GF. Whole cell protein lysates were extracted from GF of the male control, the index patient and the CAIS patient with a documented frameshift mutation and treated with 10nM DHT or ethanol, respectively. From all samples, 35 μg of protein were loaded, additionally 10 μg were loaded for the control sample to avoid overexposition. Immunoblot analysis shows a markedly reduced amount of the 112 kD full-length AR protein in the index patient and an increased detection of a shorter 75kD fragment. DHT treatment stabilizes full-length 112kD AR protein while the shorter 75kD fragment remains of similar intensity as compared to untreated GF. The corresponding bands are indicated by an arrow. An unspecific band is denoted by a star. GF from the CAIS patient served as negative control. Actin measurement served as loading control. C) DHT-dependent ectopic AR protein expression in PC3 cells. Cells were either treated with 10nM DHT or ethanol. 5′UTRmut-*AR* transfected cells show reduced AR protein expression compared to 5′UTRwt-*AR* transfected cells. AR-121 transfected cells express an AR-fragment of lower molecular weight. Actin measurement served as loading control. D) DHT-dependent activation of the AR target gene *APOD* is compromised in the index patient. AR activity was measured through the activation of the endogenous AR target gene *APOD*. This revealed a mean 3.4 fold activation of *APOD* in response to DHT stimulation in three male foreskin control derived cell lines. GF of the index patient, like GF of the CAIS patient with known mutation show a highly significant loss of *APOD* induction as compared to the male controls (p***<0.001). Means and standard deviations of three independent experiments are shown. p-values are calculated by a t-test. E) DHT-dependent activation of the AR target gene *PPAP2B* is compromised in the index patient. AR activity was measured through the activation of the endogenous AR target gene *PPAP2B*. This revealed a mean 1.54 fold activation of *PPAP2B* in response to DHT stimulation in three male foreskin control derived cell lines. GF of the index patient, like GF of the CAIS patient with known mutation show a significant loss of *PPAP2B* induction as compared to the male controls (p**<0.01). Means and standard deviations of at least three independent experiments are shown. p-values are calculated by a t-test.

The results described suggest that in patients carrying the mutation in the 5′UTR, AR function might be reduced due to reduced post-transcriptional AR protein expression. We therefore sought to analyze *AR* expression levels in GFs from the patients compared to a male control. Although we obtained GF of documented labial origin from the first patient, the second patient′s GF were taken from the inguinal region outside the genitalia which is not a classical androgen responsive tissue. We thus used only GF from patient 1. When analyzing *AR* mRNA expression no significant difference was seen between control GF and the patient′s GF (Fig 3A). At the protein level instead, the patient′s GF showed a strong reduction of the full-length AR protein. At the same time, a lower migrating band of approximately 75kD was increased in the patient′s GF compared to control GF (Fig 3B). Dihydrotestosterone (DHT) is known to activate and stabilize full length AR once it is released from the chaperones by N/C-terminal protein interaction [15]. Consistently, we found an increase of the full-length AR protein upon DHT treatment in GF from the male control and the index patient, but not of the shorter AR-fragment (Fig 3B). GF-derived protein extracts from a CAIS patient with a known AR-frameshift mutation [16] and a complete loss of AR expression served as negative control (Fig 3B).

In order to recapitulate the AR protein expression pattern *in vitro*, we transfected above described plasmids containing wild type and mutant 5`UTR in front of the *AR*-CDS into AR-depleted prostate cancer PC3 cells. We chose PC3 cells because prostate is an androgen responsive tissue and response to DHT treatment can be monitored. Indeed, *AR*5′UTRwt-*AR* transfected PC3 cells showed an enhanced AR-protein signal after DHT treatment as compared to untreated cells, indicating that the construct readily responds to androgens (Fig 3C). When looking at the *AR*5′UTRmut-*AR* construct, AR-expression levels were strongly reduced but still responsive to DHT treatment reflecting the situation in GF from patient 1 (Fig 3C). A lower molecular weight band also appears in the mutant, although at a different ratio in respect to the full length AR protein observed *in vivo*. We speculated that the lower band could be another translational start as initiation of translation at the next downstream ATG within the *AR*-CDS at amino acid 191 has been proposed in the literature and an *AR*-plasmid starting at this second ATG (AR-121) has been designed previously [17]. The calculated molecular weight of this shorter AR fragment would be 78kD. When analyzing cell extracts of PC3 cells transfected with AR-121 next to those cells transfected with the *AR*5′UTRmut-*AR* plasmid, we found that AR-121 ran at the same height as the lower molecular weight band, indicating that this band is likely to be an AR-product initiating at the second translational start (Fig 3C).

### *AR*-5′UTR mutation reduces the transcriptional activity of the AR-protein *in vivo*

Although we saw a markedly reduced AR protein expression in the index patient′s GF, it is unclear whether this reduction is sufficient to abolish the transcriptional activity of the AR hence leading to the CAIS phenotype observed in the patients. We therefore decided to test the efficiency of the altered AR protein in the index patient′s GF in inducing endogenous target gene transcription. While numerous endogenous AR target genes have been found in prostate cancer derived cell lines, only three have been described in healthy male genital skin fibroblasts [18]. Out of these, Apolipoprotein D (*APOD*) showed a reproducible DHT dependent transcriptional induction. More recently we identified another AR-target gene in GF, the Phosphatidic Acid Phosphatase Type 2B (*PPAP2B*), and included it in this study. GF derived from male foreskin tissue served as control. We measured mRNA levels of *APOD* and *PPAP2B* in the presence and absence of DHT and calculated the ratio of induction. While control GF showed a three to four fold up-regulation of *APOD* mRNA, GF of the index patient failed to respond to DHT as measured by *APOD*-induction and behaved similarly to GF obtained from a CAIS patient with a documented *AR* frameshift mutation [16] (Fig 3D). Although transcriptional induction was generally lower for *PPAP2B* compared to *APOD*, it also showed a significant induction in response to DHT in male control GF (p<0.01) but a failure of induction in GF derived from the index patient and the CAIS patient harboring the *AR* frameshift mutation (Fig 3E). We concluded that a post-transcriptional impairment of AR protein expression is responsible for the inability of DHT to induce a transcriptional active form of the AR *in vivo* and therefore confirmed the clinical diagnosis of complete androgen insensitivity at the molecular level.

### The *AR*-5′UTR uORF is translated into a polypeptide

**Fig 4:**
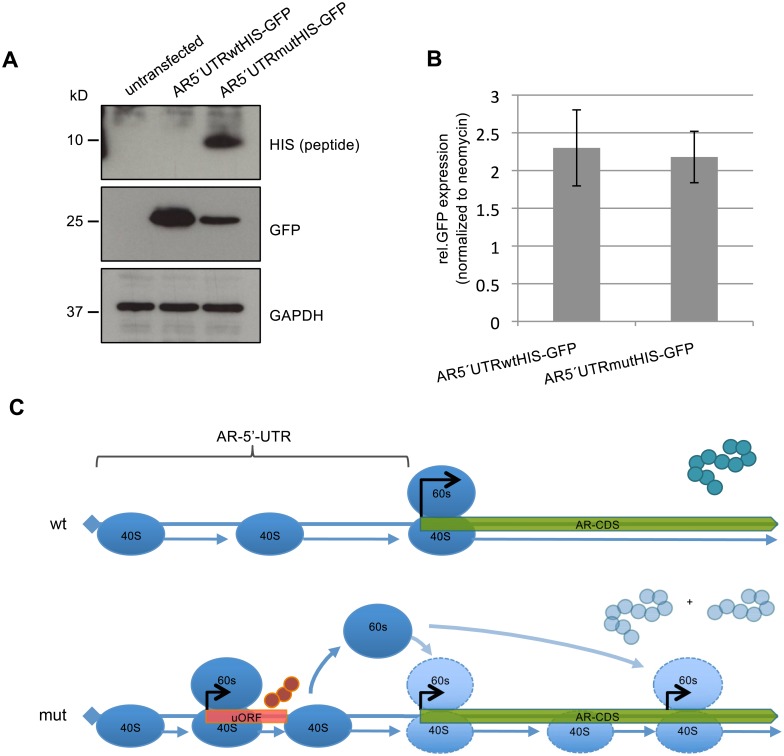
The uORF is translated into a polypeptide. Expression analysis of the HIS-tagged uORF. HEK293 cells were transfected with either *AR*5′UTRwtHIS-*GFP* or *AR*5′UTRmutHIS-*GFP*. After 48h of transfection, RNA and protein was isolated. A) GFP protein analysis. Cells transfected with the *AR*5′UTRmutHIS-*GFP* construct show a strong peptide expression and highly reduced GFP protein levels. GAPDH measurement served as loading control. The experiment was done in triplicate. One representative blot is displayed. B) Q-RT-PCR analysis of *GFP* mRNA. There is no significant difference in the *GFP* mRNA levels between the *AR*5′UTRwtHIS-*GFP* and the *AR*5′UTRmutHIS-*GFP* construct (p = 0.64). *GFP* mRNA levels were normalized to the neomycin resistance expression of the vector. Experiments were performed in triplicate. C) Model for impaired AR-translation. In the wild type (wt) situation the 40S ribosomal subunit scans the 5′UTR for the translational start codon of the AR coding sequence (CDS). The majority of ribosomal complex will form at the canonical AR-start codon and protein translation starts. In the mutant (mut) 5′UTR a full ribosomal complex is loaded at the start of the uORF generated though the c.-5467C>T mutation creating a polypeptide of 63 amino acids. Ribosome stalling and/or dissociation of the 60S subunit at the end of the uORF would cause a poor re-initiation at the AR pORF. Internal ribosomal binding may lead to re-initiation at the next available ATG within a Kozak sequence to produce a shorter (75 kD) AR protein.

Finally we set out to elucidate the mechanism by which the mutation alters AR protein levels. Two not mutually exclusive scenarios can be postulated to explain the observed results. In the first scenario, the ribosomes are stalled at the *de novo* ATG and therefore reach only insufficiently the *AR*-primary ORF (pORF). In the second option, a polypeptide is built from this ATG. The majority of ribosomes would fall off after completion of the polypeptide and only few would reach the *AR*-pORF due to leaky scanning [3]. In order to test the second option we cloned a 6xHistidine (6xHIS)-tag in front of the putative uORF stop-codon within the *AR*-5′UTR and replaced the tagged uORF version (uORF-HIS) with the untagged one in the above described *AR*5′UTR-*GFP* vector. This resulted in an *AR*5′UTRwt-HIS-*GFP* and an *AR*5′UTRmut-HIS-*GFP* vector with ATG as potential uORF start codon (S3 Fig). The new uORF of 204 nt would produce a peptide of 68 amino acids including the 6xHIS-tag and an expected molecular weight of 8.4kDa. After transfection of both constructs in HEK293 cells, we readily detected a polypeptide in the mutant tagged *AR*5′UTRmut-HIS-*GFP* vector of the expected molecular weight (Fig 4A) indicating usage of the newly introduced uORF by the translational machinery. GFP expression in both constructs showed the opposite pattern in respect to the uORF-HIS expression, evidencing that the context of the newly generated uORF start codon leads to the translation of a HIS-tagged polypeptide and suppresses the translation of the consecutive GFP protein (Fig 4A). Analyzing *GFP* mRNA levels revealed again no difference between the *AR*5′UTRwt-HIS-*GFP* and the *AR*5′UTRmut-HIS-*GFP* (Fig 4B). These results show that strong translation of the uORF encoded polypeptide within the 5′UTR of the *AR* is responsible for the reduced GFP and thus AR expression.

## Discussion

According to the McGill Androgen Receptor Mutation Database over 500 *AR* gene mutations have been identified in AIS as well as prostate cancer patients [9]. Almost all of the described mutations are located in coding exons including splice site regions. Only three mutations within the 5`-UTR have been reported so far in breast and prostate cancer, but none in AIS [19, 20]. However, a functional impact of these mutations has not been analyzed.

Using NGS covering the entire *AR* gene locus we discovered a hitherto unrecognized mutation in the 5′UTR of the *AR* to be recurrent in AIS. We show that this mutation creates a uORF encoded peptide, and that translation of the uORF suppresses protein expression of the pORF without changing mRNA expression. We therefore confirm the hypothesis of Mikozami et al. [13] who postulated in 1994 that mutations in the *AR*-5′UTR might lead to AIS.

According to common models, uORF translation into small peptides can either cause complete dissociation of the ribosomal subunits at the end of the uORF or the remaining of the 40S ribosomal subunit on the transcript and its scanning for the next available ATG [3]. The here presented case fits the second option because we see strong reduction but no complete inhibition of translation at the pORF (Figs 3B and 3C, 4A and 4C). One of the features affecting the efficiency of uORF-mediated translational repression is the intercistronic distance between the uORF and the pORF. In the case of the *AR*-5′UTR mutation, the distance might be too small for proper reassembly at the pORF but might enable reassembly at a further downstream ORF. Further experiments are planned to test if different spacing between the cistrons influence translational start site usage. In line with this, a lower molecular weight AR fragment of approximately 75 kD appears in the index patient′s GF, possibly generated by re-initiation of translation at an ATG downstream of the canonical *AR*–pORF (Figs 3B and 3C and 4C). uORF mediated switches in translational start sites have been described in the literature [21] and alternative start site usage has been postulated for the *AR* occurring at the first internal methionine 191 (S1 Fig) in patients carrying an *AR*-stop mutation before amino acid 191 [22–24]. The resulting N-terminal deleted AR protein lacks the transactivating domain [17, 25]. An intact N-terminus is crucial for an activating interaction between the N-and C-terminus of the AR [26].

After DHT induction, AR full length protein in the index patient is still detectable at a low level. Nevertheless, both patients have a complete lack of external genital virilisation. Accordingly, there is no AR transcriptional activity in the patient′s GF confirming the diagnosis on a molecular level. A possible explanation could be that the low amount of AR protein is not sufficient for the development of the male phenotype during embryogenesis. Alternatively, the shorter AR-fragment detected in the GF could act as a competitive inhibitor on AR transcriptional activity because it still has a DNA-binding but no transactivation domain. In line with this, it has been shown that the N-terminal truncated shorter AR fragment starting at methionine 191 significantly reduces activity of full length AR upon DHT treatment in *AR* transfected GF [27]. The fact that the lower molecular weight band is also visible in the control sample, although much weaker compared to the full length AR, could be a consequence of leaky ribosome scanning at the pORF. As full length AR protein levels exceed largely those of the shorter fragment it is unlikely to have an effect on AR-function.

In conclusion, we provide a novel explanation for the molecular pathogenesis of AIS, one of the most common causes of DSD. On a more general level, our results suggest that mutations altering translational control might account for a considerable number of patients displaying characteristic monogenic phenotypes of endocrine diseases and beyond but lacking protein coding mutations in the associated genes to date.

## Materials and Methods

### Sample collection, fibroblast strains and cell culture conditions

The study was performed with approval of the Ethical Committee of the Christian-Albrechts-University, Kiel, Germany (AZ: D415/11) (S3 File). We obtained written consent from the parents on behalf of the children/minors enrolled in this study. Control foreskin fibroblasts were obtained from biopsies during circumcision of males with phenotypically normal male external genitalia. Fibroblasts from patient 1 originated from labia minora tissue which is homologous to the male foreskin. Fibroblasts from a 46,XY CAIS individual with proven frameshift mutation (Pro219fsX) also originated from labia minora tissue [16]. GF were grown in phenol red free Dulbecco′s modified Eagle′s medium (Life Technologies) supplemented with 10% fetal bovine serum (FBS; Biochrome), 100 units/ml penicillin/streptomycin, 2 mM L-glutamine (Biochrome) and 20 mM HEPES buffer (Life Technologies) at 37°C with 5% CO2. For hormone induction experiments, 1.2x105 cells were plated in three 6cm dishes each and incubated at 37°C with 5% CO2. After 24h, time point zero was taken by collecting the cells in RNA-extraction buffer (RLT; Qiagen). The remaining two dishes were washed three times in the above medium without FBS and further grown in the same medium supplemented with 0.1% charcoal treated FBS (serum starved conditions). To the first dish DHT (Sigma-Aldrich, Germany) dissolved in 100% ethanol was added to a final concentration of 10 nM. An equal volume of ethanol was added to the second dish. Cells were left for 72h under these conditions at 37°C with 5% CO2 after which they were lysed in RNA-extraction buffer (RLT; Qiagen). For protein extractions, cells were grown in complete tissue culture medium containing 10% charcoal treated FBS. For hormone treatment, DHT was added to a final concentration of 10nM. Protein lysates were taken 72h after hormone treatment.

### Next generation sequencing library preparation and sequencing

The sequencing library was prepared from genomic DNA of both index patients as well as controls according to the Haloplex protocol (Agilent) with oligos spanning the chromosomal region ChrX:66,754,874–66,955,461 (hg19). Sequencing was performed on a MiSeq benchtop sequencer (Illumina). Alignment to the hg19 reference genome and single nucleotide polymorphism (SNP) was performed by the MiSeq-Reporter software (Illumina). Small insertion deletion (indel) calling was performed using the SureCall software 3.0.1.4 from Agilent.

Sanger sequencing was performed on ABI 310 and ABI 3130 Genetic Analyzers (Applied Biosystems) according to standard protocols.

### Cloning of the 5′UTR of the *AR*

In order to investigate the effect of the *AR*-5’UTR on the expression of GFP we created a modified version of the pcDNA3.1 plasmid by removing all transcribed sequences between the putative transcription start site (TSS) and the polyadenylation signal [28]. The vector was created by PCR using primers that were able to amplify the pcDNA3.1+ backbone and concomitantly inserted an EcoRI and a BamHI site downstream of the TSS. Two PCR fragments corresponding to the 5′UTR of the *AR* (generated from genomic DNA of a male control and of patient 1) and to the *GFP* gene were created with primers inserting an EcoRI and NcoI site to the wt and mutant *AR*-5’UTR, and a NcoI and BamHI site to GFP. The two fragments were ligated into the modified pcDNA3.1 vector and sequenced to verify the correct sequence. The uORF is out of frame related to the *GFP* gene, reflecting the *in vivo* situation of the *AR*. For cloning of the *AR*-5’UTR in front of the *AR*-CDS, we performed a PCR covering the *AR*-5’UTR inserting a NsiI and BamhHI site at the very 5′ end until the SexAI restriction site at the beginning of the *AR*-CDS. This PCR product was cut with NsiI and SexAI and cloned into a NsiI/ SexAI cut psvARO vector [17]. A BamHI fragment covering the entire *AR*-5′UTR and -CDS was then cloned into the above pcDNA3.1 derived vector and sequenced for verification. For the HIS tagged uORF an overlap extension PCR was performed in order to insert six histidines in front of the uORF stop codon in both the wt and mut *AR*-5′UTR. The HIS-tagged uORF containing *AR*-5′UTR was then cloned in the modified pcDNA3.1 vector and sequenced as described above. A schematic representation of the *in vitro* constructs is shown in S3 Fig. Primers used for cloning are listed in S1 Table.

### Transfection experiments

HEK293T cells were grown in DMEM supplemented with 10% FBS. Typically 400,000 cells were plated in each well of a 6 well plate and transfected the day after using 1 μg of plasmid DNA/well and lipofectamine 2000 (Life Technologies). After 72h of transfection, cells were collected for concomitant protein and RNA extraction using the PARIS kit (Life Technologies) and for FACS analysis by resuspending the cells in phosphate buffered saline (PBS) supplemented with 2% FBS. For luciferase experiments 200,000 HEK293T cells were transfected with 10 ng of either *AR*5′UTRwt-*AR* vector, *AR*5′UTRmut-*AR* vector or empty vector together with 80 ng Renilla-luciferase vector as well as 400 ng of the ARE-luciferase reporter vector. The next day, DHT was added to the cells at a final concentration of 10 nM in order to activate the AR-protein. After further 24h, Firefly luciferase and Renilla luciferase expressions were measured according to the Dual-Luciferase Reporter Assay protocol (Promega) using a Veritas microplate luminometer (Turner BioSystems). PC3 cells were grown in RPMI supplemented with 10% FBS. Typically 200,000 cells were plated in each well of a 6 well plate and transfected the day after using 2 μg of plasmid DNA/well and lipofectamine 2000 (Life Technologies). The next day, cells were washed twice in RPMI without FBS and then incubated in RPMI supplemented with charcoal treated FBS containing either 10nM DHT or 100% ethanol for 24 hours. Cells were lysed in RIPA buffer for protein extraction.

### FACS analysis

Roughly 1x10^5^ cells were resuspended in FACS buffer (PBS, 2% FBS). Flow cytometry data were acquired on a BD Accuri C6 (BD Biosciences) and analyzed with the BDCSampler software (BD Biosciences).

### RNA isolation, amplification and detection

Total RNA was isolated from fibroblasts using the RNeasy kit (Qiagen). 500ng of total RNA was reverse transcribed using the QuantiTect Reverse Transcription Kit (Qiagen). Quantitative PCR was performed with the QuantiTect SYBR Green master mix (Qiagen) using primers against *APOD*, *AR* and *SDHA*. All primers were purchased from Qiagen and used following the manufacturer′s instructions. For HEK293T transfections, 500ng RNA extracted using the PARIS kit (Life Technologies) was treated twice with TURBO DNase (Life Technologies) to eliminate residual plasmid DNA and reverse transcribed using the QuantiTect Reverse Transcription Kit (Qiagen). The reverse transcription reaction was also performed in the absence of Reverse Transcriptase. Quantitative PCR was performed with the QuantiTect SYBR Green master mix (Qiagen) using primers against the *AR*-5′UTR and *GFP* (S1 Table) and the neomycin resistance gene (neo) [29]. Primer were designed using the Primer3 software. p-values were calculated using a two-sided t-test.

### Protein isolation and detection

Whole cell protein lysates were obtained by resuspending the cells in ice-cold RIPA buffer, supplemented with a cocktail of protease inhibitors (CompleteTM, Roche). Protein extracts were separated by SDS-PAGE on a NuPAGE^®^ Novex^®^ 4–12% Bis-Tris Protein gel (Life Technologies) and transferred to a nitrocellulose membrane (Whatman, GE Healtehcare Life Sciences). The membrane was blocked for 1-2h at room temperature with TBS containing 0.1% Tween and 5% non-fat dry milk. For AR and HIS detection F39.4.1 antibody (BioGenex) (1:100 dilution) and Anti-6xHIS tag antibody (Anti-HIS HRP, MACS) (1:10,000 dilution) was used respectively over night at 4°C, for actin detection Anti-Actin (A 2066) antibody (Sigma-Aldrich) was used for 1-2h at room temperature (1:10,000 dilution). Anti-GFP-HRP (MACS) was used for the duration of 1h at room temperature for GFP detection (1:5,000 dilution) and GAPDH antibody (GeneTex) was used for 45min at room temperature (1:10,000 dilution) for GAPDH detection. Secondary anti-rabbit and anti-mouse antibodies (Immuno Reagents Inc.) were incubated for 1-2h at room temperature (1:4,000 dilution). All antibodies were resuspended in TBS containing 0.1% Tween and 5% non-fat dry milk. Signals were detected on an Amersham Hyperfilm ECL (GE Healthcare) using the Luminata Forte Western HRP Substrate (Millipore). Expected molecular weights of polypeptides were calculated using EMBOSS GUI v.1.14: pepstats.

## Supporting Information

S1 Fig Kozak sequences around the start codon of the uORF, pORF and the downstream ORF starting at amino acid 191 of the AR. The depicted Kozak consensus sequence is a sequence occurring on eukaryotic mRNA. Big letters correspond to high evolutionary conservation.
(PDF)

S2 Fig PCR analysis of GFP and neomycin resistance mRNA after DNAse treatment. HEK293 cells were transfected with either empty vector, *AR*5′-UTRwt-*GFP*, *AR*5′-UTRmut-*GFP* or not transfected at all. After 72h of transfection, RNA was isolated and treated with DNAse in order to completely digest vector DNA. The expected size for neo is 95bp, that of GFP is 201bp. The minus reverse transcriptase control (-RT) shows no PCR-product, demonstrating that plasmid-DNA was completely digested from the samples.
(PDF)

S3 Fig Schematic representation of the constructs used for the *in vitro* studies. (PDF)

S1 File List of SNPs found in patient 1. (XLSX)

S2 File List if SNPs found in patient 2. (XLSX)

S3 File Study approval by the Ethical Committee of the Christian-Albrechts-University, Kiel, Germany. (PDF)

S1 Table List of primers used in this study. (PDF)
