# Supplementary material for: A Recurrent Germline Mutation in the 5’UTR of the Androgen Receptor Causes Complete Androgen Insensitivity by Activating Aberrant uORF Translation
Source: PLoS One. 2016 Apr 25;11(4):e0154158. doi: 10.1371/journal.pone.0154158 (PMC4844194; doi:10.1371/journal.pone.0154158)
Supplement: S1 Fig — The depicted Kozak consensus sequence is a sequence occurring on eukaryotic mRNA. Big letters correspond to high evolutionary conservation. (PDF) [file pone.0154158.s001.pdf]

Kozak consensus sequence	**A/G C C A T G G C/A**
wt - uORF Kozak sequence	**G C C A C G A C**
mut - uORF Kozak sequence	**G C C A T G A C**
pORF Kozak sequence	**A G G A T G G A**
Downstream ORF Kozak sequence (aa 191)	**A C C A T G C A**
