# Supplementary figures and images for: A Recurrent Germline Mutation in the 5’UTR of the Androgen Receptor Causes Complete Androgen Insensitivity by Activating Aberrant uORF Translation
Source: PLoS One. 2016 Apr 25;11(4):e0154158. doi: 10.1371/journal.pone.0154158 (PMC4844194; doi:10.1371/journal.pone.0154158)

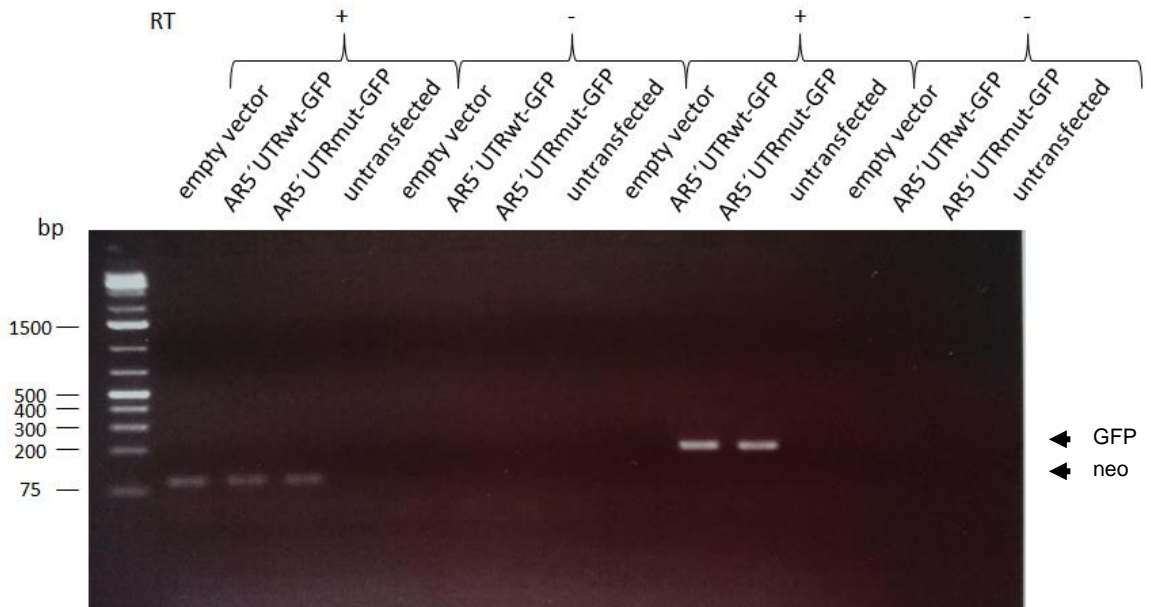

Supplement: S2 Fig — HEK293 cells were transfected with either empty vector, AR5′-UTRwt-GFP, AR5′-UTRmut-GFP or not transfected at all. After 72h of transfection, RNA was isolated and treated with DNAse in order to completely digest vector DNA. The expected size for neo is 95bp, that of GFP is 201bp. The minus reverse transcriptase control (-RT) shows no PCR-product, demonstrating that plasmid-DNA was completely digested from the samples. (PDF) [file pone.0154158.s002.pdf]

AR5'UTR

↓

a)

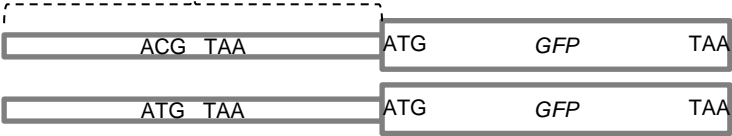

AR5'UTRwt-GFP

AR5'UTRmut-GFP

b)

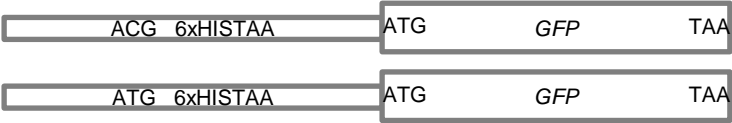

AR5'UTRwtHIS-GFP

AR5'UTRmutHIS-GFP

c)

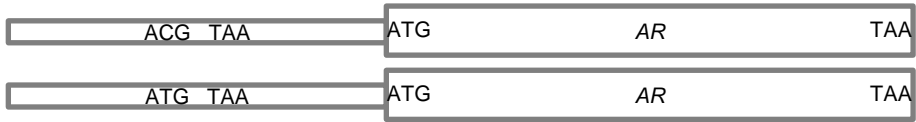

AR5'UTRwt-CDS

AR5'UTRmut-CDS

Supplement: S3 Fig — (PDF) [file pone.0154158.s003.pdf]
