# Supplementary material for: A Recurrent Germline Mutation in the 5’UTR of the Androgen Receptor Causes Complete Androgen Insensitivity by Activating Aberrant uORF Translation
Source: PLoS One. 2016 Apr 25;11(4):e0154158. doi: 10.1371/journal.pone.0154158 (PMC4844194; doi:10.1371/journal.pone.0154158)
Supplement: S3 File — (PDF) [file pone.0154158.s006.pdf]

MEDIZINISCHE FAKULTÄT
DER CHRISTIAN-ALBRECHTS-UNIVERSITÄT ZU KIEL

ETHIK-KOMMISSION

28. MÄR. 2011 Eing

A.D. 1665

Universitäts-Kinderklinik · Schwanenweg 20 · 24105 Kiel

Prof. Dr. med. P.-M. Holterhus
Klinik für Allgemeine Pädiatrie
Endokrinologische Ambulanz
UKSH, Campus Kiel
Arnold-Heller-Str. 3, Haus 9
24105 Kiel

Schwanenweg 20
D-24105 Kiel

Telefon 04 31 / 597-18 09
Telefax 04 31 / 597-18 31

Datum:

16.03.2011

AZ.: D 415/11 (bitte stets angeben)

Studienplan: **Androgenresistenz ohne Androgenrezeptorgenmutation: von der funktionellen Charakterisierung zum Epigenotyp**
(in Bezug auf AZ. A 410/08: Investigation of the molecular pathogenesis and patho- physiology of Disorders of Sex Development (DSD) –EuroDSD)
DFG-Sachbeihilfeantrag

Ihr Schreiben vom: **04.03.2011 (Eingang 07.03.2011)**

Lieber Herr Holterhus,

vielen Dank für Ihren obengenannten Antrag zur Beratung gemäß § 15 Berufsordnung (BO) der Ärztekammer Schleswig-Holstein.

Nach Durchsicht der Unterlagen durch die Geschäftsstelle und durch mich als Vorsitzenden der Ethik-Kommission bestehen gegen die Durchführung des Projektes keine berufsethischen und berufsrechtlichen Bedenken.

Mit freundlichen kollegialen Grüßen

Prof. Dr. med. H. M. Mehdorn
Vorsitzender der Ethik-Kommission

Dr. med. Christine Glinicke
Geschäftsführung der Ethik-Kommission
