# Supplementary material for: A Recurrent Germline Mutation in the 5’UTR of the Androgen Receptor Causes Complete Androgen Insensitivity by Activating Aberrant uORF Translation
Source: PLoS One. 2016 Apr 25;11(4):e0154158. doi: 10.1371/journal.pone.0154158 (PMC4844194; doi:10.1371/journal.pone.0154158)
Supplement: S1 Table — (PDF) [file pone.0154158.s007.pdf]

Primer used for cloning	
TSSAS1	5´CGGAATTCTCTCTAGTTAGCCAGAGAGC3´
BGHS1	5´CGGAATTCATAGGATCCTTTCCTAATAAAATGAGGAAATTGC3´
GFP-S1	5´CATGCCATGGTGAGCAAGGGCGAGG3´
GFP-AS1	5´GCGGATCCTTACTTGTACAGCTCGTCC3´
AR5´UTR-S1	5´CGGAATTCCGAGATCCCGGGGAGCCAG3´
AR5´UTR-AS1	5´CAGATGCATGGATCCGAGATCCCGGGGAG3´
NsiI-BamHI-AR5´UTR-S	5´TTCCTCATCCAGGACCAGGTAGCCTGT3´
SexAI-AR-AS	5´CATGCCATGGTTGAGCTTGGCTGAATCTTCC3´
AR5UTR-HIS-S	5´CACCACCACCACCACCACTAATAACTCAGTTCTTATTTGC3´
AR5UTR-HIS-AS	5´GTGGTGGTGGTGGTGGTGTCTTTTTCTTTTATTTCGGC3´
Primer used for qPCR	
GFP fw	5´AAGCTGACCCTGAAGTTCATCTGC3´
GFP rev	5´CTTGTAGTTGCCGTCGTCCTTGAA3´
ARseq4-up	5´GAGGCGACAGAGGGAAAAAG3´
ARseq4-do	5´TTGGAAGGTGGAGGATTTT3´
ARseq4a-up	5´AGCTGCACATTGCAAAGAAG3´
ARseq4a-do	5´GAAAAGGCAGTCAGGTCTTCA3´
